# {3,3′,5,5′-Tetra­meth­oxy-2,2′-[ethane-1,2-diylbis(nitrilo­methyl­idyne)]diphenol­ato}­copper(II)

**DOI:** 10.1107/S1600536810017137

**Published:** 2010-05-15

**Authors:** Gervas Assey, Ray J. Butcher, Yilma Gultneh

**Affiliations:** a Department of Chemistry, Howard University, 525 College Street NW, Washington, DC 20059, USA

## Abstract

In the title square-planar copper complex, [Cu(C_20_H_22_N_2_O_6_)], the Cu—N and Cu—O bond lengths are significantly longer than those of its isostructural nickel analog. The title structure is related to that of the corresponding monohydrate. There are significant differences in the conformations of the two complexes. While the monohydrate is mainly planar, in the title compound there is a slight twist in the two benzene rings at each end of the complex [dihedral angle = 13.14 (6)°]. All the atoms of the meth­oxy substitutents are in the plane of the ring to which they are attached (r.m.s. deviation = 0.0079 Å) except for one of the meth­oxy C atoms, which deviates slightly [0.309 (4) Å]. In the crystal, weak C—H⋯O inter­molecular inter­actions link the mol­ecules.

## Related literature

For similar Cu–salen {salen is 2,2′-[ethane-1,2-diylbis(nitrilo­methylidyne)]diphenolate}complexes, see: Labisbal *et al.* (1994 ▶). For the isostructural nickel analog, see: Assey *et al.* (2010 ▶). 
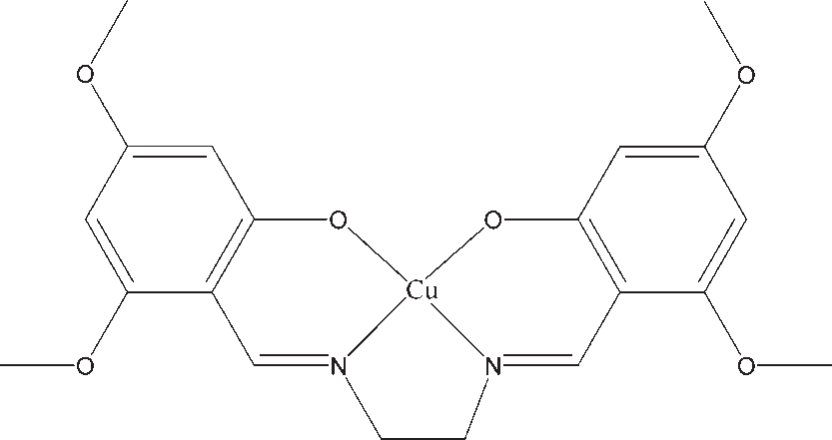


## Experimental

### 

#### Crystal data

[Cu(C_20_H_22_N_2_O_6_)]
*M* *_r_* = 449.94
Monoclinic, 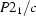

*a* = 7.3953 (2) Å
*b* = 15.8514 (5) Å
*c* = 15.7042 (4) Å
β = 91.842 (3)°
*V* = 1839.97 (10) Å^3^
*Z* = 4
Cu *K*α radiation
μ = 2.06 mm^−1^
*T* = 110 K
0.51 × 0.29 × 0.25 mm

#### Data collection

Oxford Diffraction Xcalibur diffractometer with a Ruby (Gemini Cu) detector
Absorption correction: multi-scan (*CrysAlis PRO*; Oxford Diffraction, 2009 ▶) *T*_min_ = 0.281, *T*_max_ = 1.000
7277 measured reflections
3608 independent reflections
3370 reflections with *I* > 2σ(*I*)
*R*_int_ = 0.023

#### Refinement

*R*[*F* ^2^ > 2σ(*F* ^2^)] = 0.036
*wR*(*F* ^2^) = 0.103
*S* = 1.04
3608 reflections
266 parameters
H-atom parameters constrained
Δρ_max_ = 0.44 e Å^−3^
Δρ_min_ = −0.67 e Å^−3^

### 

Data collection: *CrysAlis PRO* (Oxford Diffraction, 2009 ▶); cell refinement: *CrysAlis PRO*; data reduction: *CrysAlis PRO*; program(s) used to solve structure: *SHELXS97* (Sheldrick, 2008 ▶); program(s) used to refine structure: *SHELXL97* (Sheldrick, 2008 ▶); molecular graphics: *SHELXTL* (Sheldrick, 2008 ▶); software used to prepare material for publication: *SHELXTL*.

## Supplementary Material

Crystal structure: contains datablocks I, global. DOI: 10.1107/S1600536810017137/jj2031sup1.cif

Structure factors: contains datablocks I. DOI: 10.1107/S1600536810017137/jj2031Isup2.hkl

Additional supplementary materials:  crystallographic information; 3D view; checkCIF report

## Figures and Tables

**Table 1:** Selected bond lengths (Å)

Cu—O1	1.9059 (13)
Cu—O2	1.9070 (13)
Cu—N2	1.9314 (16)
Cu—N1	1.9347 (15)

**Table 2:** Hydrogen-bond geometry (Å, °)

*D*—H⋯*A*	*D*—H	H⋯*A*	*D*⋯*A*	*D*—H⋯*A*
C18—H18*A*⋯O5^i^	0.98	2.57	3.439 (2)	148

Symmetry code: (i) 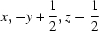.

**Fig. 1.**
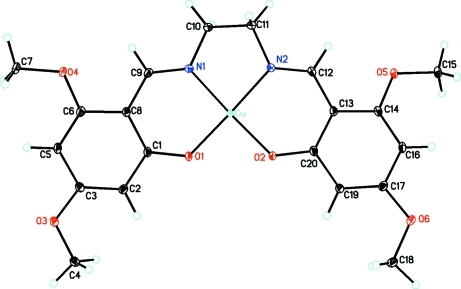
Diagram of the square planar copper complex C20H22N2CuO6 showing atom labeling.

**Fig. 2.**
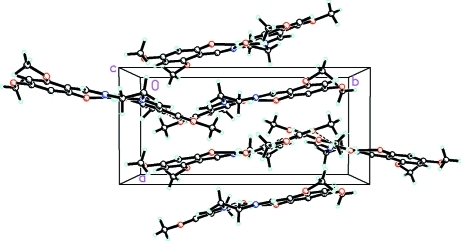
The molecular packing for C20H22N2CuO6 viewed down the c axis.

## supplementary crystallographic information

### Comment

The structure is reported of the square planar copper complex C_20_H_22_N_2_CuO_6_, which is related a previously published structure, which crystallized as a monohydrate (Labisbal *et al.*, 1994).

The Cu—N and Cu—O bond distances of 1.9314 (16) Å, 1.9347 (15) and 1.9059 (13) Å, 1.9070 (13) Å are significantly longer than those found in both the polymorph [1.892 (3) and 1.898 (3) \%A for Cu—O and 1.908 (4) and 1.912 (4) \%A for Cu—N], and the structure of the isotructural nickel derivative (Assey *et al.*, 2010). There are significant differences in the conformations of the structures. While the monohydrate is mainly planar, in the title compound there is a slight twist in the two phenyl rings at each end of the complex (dihedral angle of 13.14 (6)°). All the atoms of the methoxy substitutents are in the plane of the ring to which they are attached except C7 which deviates slightly [0.309 (4) °]. There are weak C—H···O intermolecular interactions which link the molecules.

### Experimental

The ligand synthesis was accomplished by adding a solution of (2 g, 33.3 mmol) ethylenediamine in 25 ml of methanol to a solution of (12.13 g, 66.6 mmol) 4,6-dimethoxysalicylaldehyde in 40 ml of methanol. The mixture was refluxed overnight while stirring. Then the mixture was evaporated under reduced pressure to afford yellow solids. The complex was synthesized by mixing a solution of (0.38 g, 1 mmol) *N*,*N*-ethylenebis(4,6-dimethoxysalicylaldimine) in 5 ml of CH_2_Cl_2_ with a solution of (0.29 g, 1 mmol) copper acetate in 5 ml methanol. The solution mixture was stirred for 1 hour then filtered and layered with diethyl ether for crystallization. Single crystals of X-ray quality were obtained.

### Refinement

H atoms were placed in geometrically idealized positions and constrained to ride on their parent atoms with a C—H distances of 0.95 and 0.99 Å *U*_iso_(H) = 1.2*U*_eq_(C) and 0.98 Å for CH_3_ [*U*_iso_(H) = 1.5*U*_eq_(C)].

### Crystal data

**Table:**

[Cu(C_20_H_22_N_2_O_6_)]	*F*(000) = 932
*M**_r_* = 449.94	*D*_x_ = 1.624 Mg m^−^^3^
Monoclinic, *P*2_1_/*c*	Cu *K*α radiation, λ = 1.54184 Å
Hall symbol: -P 2ybc	Cell parameters from 5963 reflections
*a* = 7.3953 (2) Å	θ = 5.6–73.9°
*b* = 15.8514 (5) Å	µ = 2.06 mm^−^^1^
*c* = 15.7042 (4) Å	*T* = 110 K
β = 91.842 (3)°	Thick needle, pale red-brown
*V* = 1839.97 (10) Å^3^	0.51 × 0.29 × 0.25 mm
*Z* = 4	

### Data collection

**Table:**

Oxford Diffraction Xcalibur diffractometer with a Ruby (Gemini Cu) detector	3608 independent reflections
Radiation source: Enhance (Cu) X-ray Source	3370 reflections with *I* > 2σ(*I*)
graphite	*R*_int_ = 0.023
Detector resolution: 10.5081 pixels mm^-1^	θ_max_ = 74.0°, θ_min_ = 5.6°
ω scans	*h* = −8→8
Absorption correction: multi-scan (*CrysAlis PRO*; Oxford Diffraction, 2009)	*k* = −16→19
*T*_min_ = 0.281, *T*_max_ = 1.000	*l* = −19→19
7277 measured reflections	

### Refinement

**Table:**

Refinement on *F*^2^	Primary atom site location: structure-invariant direct methods
Least-squares matrix: full	Secondary atom site location: difference Fourier map
*R*[*F*^2^ > 2σ(*F*^2^)] = 0.036	Hydrogen site location: inferred from neighbouring sites
*wR*(*F*^2^) = 0.103	H-atom parameters constrained
*S* = 1.04	*w* = 1/[σ^2^(*F*_o_^2^) + (0.0699*P*)^2^ + 1.2928*P*] where *P* = (*F*_o_^2^ + 2*F*_c_^2^)/3
3608 reflections	(Δ/σ)_max_ = 0.001
266 parameters	Δρ_max_ = 0.44 e Å^−^^3^
0 restraints	Δρ_min_ = −0.67 e Å^−^^3^

### Special details

**Table:**

Experimental. CrysAlisPro, Oxford Diffraction Ltd., Version 1.171.33.34d (release 27-02-2009 CrysAlis171 .NET) (compiled Feb 27 2009,15:38:38) Empirical absorption correction using spherical harmonics, implemented in SCALE3 ABSPACK scaling algorithm.
Geometry. All esds (except the esd in the dihedral angle between two l.s. planes) are estimated using the full covariance matrix. The cell esds are taken into account individually in the estimation of esds in distances, angles and torsion angles; correlations between esds in cell parameters are only used when they are defined by crystal symmetry. An approximate (isotropic) treatment of cell esds is used for estimating esds involving l.s. planes.
Refinement. Refinement of *F*^2^ against ALL reflections. The weighted *R*-factor wR and goodness of fit *S* are based on *F*^2^, conventional *R*-factors *R* are based on *F*, with *F* set to zero for negative *F*^2^. The threshold expression of *F*^2^ > σ(*F*^2^) is used only for calculating *R*-factors(gt) etc. and is not relevant to the choice of reflections for refinement. *R*-factors based on *F*^2^ are statistically about twice as large as those based on *F*, and *R*- factors based on ALL data will be even larger.

### Fractional atomic coordinates and isotropic or equivalent isotropic displacement parameters (Å^2^)

**Table:**

	*x*	*y*	*z*	*U*_iso_*/*U*_eq_	
Cu	0.25447 (4)	0.525237 (16)	0.433936 (15)	0.01235 (12)	
O1	0.25593 (19)	0.64183 (8)	0.40431 (8)	0.0170 (3)	
O2	0.2540 (2)	0.49500 (9)	0.31640 (8)	0.0177 (3)	
O3	0.1266 (2)	0.93385 (9)	0.42484 (9)	0.0216 (3)	
O4	0.09313 (19)	0.76058 (9)	0.67036 (8)	0.0181 (3)	
O5	0.45901 (18)	0.21001 (8)	0.34323 (8)	0.0164 (3)	
O6	0.3678 (2)	0.33149 (9)	0.07068 (8)	0.0190 (3)	
N1	0.2340 (2)	0.54910 (11)	0.55406 (10)	0.0145 (3)	
N2	0.2733 (2)	0.40900 (10)	0.47001 (9)	0.0142 (3)	
C1	0.2100 (2)	0.70552 (12)	0.45207 (11)	0.0137 (3)	
C2	0.1913 (2)	0.78495 (12)	0.41203 (11)	0.0153 (4)	
H2A	0.2113	0.7902	0.3528	0.018*	
C3	0.1442 (3)	0.85471 (12)	0.45847 (12)	0.0162 (4)	
C4	0.1614 (3)	0.94303 (12)	0.33597 (13)	0.0219 (4)	
H4A	0.1500	1.0025	0.3198	0.033*	
H4B	0.0739	0.9094	0.3023	0.033*	
H4C	0.2842	0.9234	0.3251	0.033*	
C5	0.1081 (3)	0.84984 (12)	0.54622 (12)	0.0172 (4)	
H5A	0.0734	0.8986	0.5769	0.021*	
C6	0.1246 (2)	0.77302 (12)	0.58594 (12)	0.0153 (4)	
C7	0.0026 (3)	0.82691 (12)	0.71463 (12)	0.0181 (4)	
H7A	−0.0319	0.8066	0.7708	0.027*	
H7B	−0.1061	0.8440	0.6817	0.027*	
H7C	0.0841	0.8754	0.7216	0.027*	
C8	0.1771 (2)	0.69860 (12)	0.54123 (11)	0.0141 (4)	
C9	0.1990 (2)	0.62182 (12)	0.58746 (11)	0.0144 (4)	
H9A	0.1870	0.6241	0.6475	0.017*	
C10	0.2694 (3)	0.47571 (11)	0.60918 (12)	0.0162 (4)	
H10A	0.1957	0.4792	0.6606	0.019*	
H10B	0.3987	0.4741	0.6275	0.019*	
C11	0.2201 (3)	0.39663 (13)	0.55836 (11)	0.0167 (4)	
H11A	0.2838	0.3471	0.5833	0.020*	
H11B	0.0882	0.3863	0.5600	0.020*	
C12	0.3216 (2)	0.34537 (11)	0.42416 (12)	0.0139 (3)	
H12A	0.3390	0.2925	0.4517	0.017*	
C13	0.3503 (2)	0.34984 (11)	0.33470 (11)	0.0135 (4)	
C14	0.4107 (2)	0.27605 (11)	0.29156 (12)	0.0135 (3)	
C15	0.5160 (3)	0.13421 (12)	0.30225 (12)	0.0188 (4)	
H15A	0.5576	0.0934	0.3454	0.028*	
H15B	0.6152	0.1472	0.2644	0.028*	
H15C	0.4143	0.1103	0.2688	0.028*	
C16	0.4194 (2)	0.27244 (12)	0.20436 (12)	0.0151 (4)	
H16A	0.4629	0.2233	0.1771	0.018*	
C17	0.3626 (2)	0.34309 (12)	0.15641 (11)	0.0148 (4)	
C18	0.3346 (3)	0.40405 (13)	0.01816 (12)	0.0241 (4)	
H18A	0.3353	0.3875	−0.0419	0.036*	
H18B	0.4292	0.4462	0.0296	0.036*	
H18C	0.2164	0.4282	0.0309	0.036*	
C19	0.3084 (3)	0.41683 (12)	0.19463 (11)	0.0147 (4)	
H19A	0.2738	0.4640	0.1605	0.018*	
C20	0.3042 (2)	0.42257 (11)	0.28476 (12)	0.0137 (3)	

### Atomic displacement parameters (Å^2^)

**Table:**

	*U*^11^	*U*^22^	*U*^33^	*U*^12^	*U*^13^	*U*^23^
Cu	0.01952 (18)	0.00796 (17)	0.00974 (17)	0.00107 (9)	0.00301 (11)	−0.00065 (9)
O1	0.0278 (7)	0.0090 (6)	0.0146 (6)	0.0013 (5)	0.0072 (5)	−0.0015 (5)
O2	0.0322 (8)	0.0100 (6)	0.0109 (6)	0.0046 (5)	0.0015 (5)	−0.0010 (5)
O3	0.0366 (8)	0.0101 (6)	0.0186 (7)	0.0025 (6)	0.0079 (6)	0.0007 (5)
O4	0.0285 (7)	0.0139 (6)	0.0120 (6)	0.0037 (5)	0.0051 (5)	−0.0032 (5)
O5	0.0241 (7)	0.0095 (6)	0.0157 (6)	0.0039 (5)	0.0020 (5)	−0.0023 (5)
O6	0.0298 (8)	0.0162 (7)	0.0113 (6)	0.0003 (5)	0.0041 (5)	−0.0029 (5)
N1	0.0202 (8)	0.0125 (8)	0.0107 (7)	0.0006 (6)	0.0007 (6)	0.0016 (6)
N2	0.0197 (8)	0.0105 (7)	0.0124 (7)	0.0019 (6)	0.0027 (6)	0.0008 (6)
C1	0.0160 (8)	0.0103 (8)	0.0151 (8)	−0.0011 (6)	0.0023 (7)	−0.0028 (7)
C2	0.0192 (9)	0.0135 (9)	0.0135 (8)	−0.0025 (7)	0.0046 (7)	−0.0011 (7)
C3	0.0204 (9)	0.0099 (9)	0.0184 (9)	−0.0010 (7)	0.0022 (7)	0.0003 (7)
C4	0.0316 (11)	0.0138 (9)	0.0210 (10)	0.0044 (8)	0.0104 (8)	0.0048 (7)
C5	0.0229 (10)	0.0119 (9)	0.0171 (9)	0.0013 (7)	0.0032 (7)	−0.0044 (7)
C6	0.0163 (9)	0.0152 (9)	0.0145 (8)	−0.0014 (7)	0.0022 (7)	−0.0035 (7)
C7	0.0217 (9)	0.0174 (9)	0.0153 (8)	0.0026 (7)	0.0045 (7)	−0.0055 (7)
C8	0.0176 (9)	0.0105 (8)	0.0143 (8)	−0.0005 (7)	0.0018 (7)	−0.0019 (7)
C9	0.0166 (9)	0.0160 (9)	0.0106 (8)	−0.0002 (7)	0.0015 (6)	−0.0026 (7)
C10	0.0235 (10)	0.0140 (9)	0.0112 (8)	0.0021 (7)	0.0006 (7)	0.0012 (6)
C11	0.0241 (10)	0.0132 (9)	0.0129 (8)	0.0011 (7)	0.0045 (7)	0.0019 (7)
C12	0.0168 (9)	0.0090 (8)	0.0159 (8)	0.0012 (6)	0.0005 (7)	0.0000 (7)
C13	0.0163 (8)	0.0097 (8)	0.0145 (8)	−0.0005 (6)	0.0013 (7)	−0.0022 (7)
C14	0.0133 (8)	0.0097 (8)	0.0175 (8)	−0.0013 (6)	0.0011 (6)	−0.0019 (7)
C15	0.0242 (10)	0.0115 (9)	0.0207 (9)	0.0049 (7)	0.0025 (7)	−0.0044 (7)
C16	0.0169 (9)	0.0104 (8)	0.0181 (9)	−0.0010 (7)	0.0036 (7)	−0.0049 (7)
C17	0.0163 (9)	0.0161 (9)	0.0124 (8)	−0.0038 (7)	0.0037 (6)	−0.0026 (7)
C18	0.0412 (13)	0.0190 (10)	0.0122 (8)	−0.0058 (9)	0.0045 (8)	0.0005 (7)
C19	0.0202 (9)	0.0109 (8)	0.0132 (8)	−0.0024 (7)	0.0024 (7)	−0.0003 (7)
C20	0.0155 (9)	0.0101 (8)	0.0157 (8)	−0.0012 (6)	0.0015 (6)	−0.0019 (7)

### Geometric parameters (Å, °)

**Table:**

Cu—O1	1.9059 (13)	C6—C8	1.433 (2)
Cu—O2	1.9070 (13)	C7—H7A	0.9800
Cu—N2	1.9314 (16)	C7—H7B	0.9800
Cu—N1	1.9347 (15)	C7—H7C	0.9800
O1—C1	1.309 (2)	C8—C9	1.424 (3)
O2—C20	1.309 (2)	C9—H9A	0.9500
O3—C3	1.366 (2)	C10—C11	1.524 (3)
O3—C4	1.435 (2)	C10—H10A	0.9900
O4—C6	1.368 (2)	C10—H10B	0.9900
O4—C7	1.438 (2)	C11—H11A	0.9900
O5—C14	1.365 (2)	C11—H11B	0.9900
O5—C15	1.433 (2)	C12—C13	1.429 (3)
O6—C17	1.360 (2)	C12—H12A	0.9500
O6—C18	1.432 (2)	C13—C20	1.429 (3)
N1—C9	1.296 (3)	C13—C14	1.430 (2)
N1—C10	1.469 (2)	C14—C16	1.374 (3)
N2—C12	1.296 (2)	C15—H15A	0.9800
N2—C11	1.467 (2)	C15—H15B	0.9800
C1—C2	1.412 (3)	C15—H15C	0.9800
C1—C8	1.433 (2)	C16—C17	1.406 (3)
C2—C3	1.376 (3)	C16—H16A	0.9500
C2—H2A	0.9500	C17—C19	1.379 (3)
C3—C5	1.414 (3)	C18—H18A	0.9800
C4—H4A	0.9800	C18—H18B	0.9800
C4—H4B	0.9800	C18—H18C	0.9800
C4—H4C	0.9800	C19—C20	1.420 (2)
C5—C6	1.372 (3)	C19—H19A	0.9500
C5—H5A	0.9500		
			
O1—Cu—O2	90.42 (6)	N1—C9—C8	125.09 (16)
O1—Cu—N2	174.72 (6)	N1—C9—H9A	117.5
O2—Cu—N2	92.41 (6)	C8—C9—H9A	117.5
O1—Cu—N1	92.85 (7)	N1—C10—C11	107.92 (15)
O2—Cu—N1	174.37 (7)	N1—C10—H10A	110.1
N2—Cu—N1	84.71 (7)	C11—C10—H10A	110.1
C1—O1—Cu	127.19 (12)	N1—C10—H10B	110.1
C20—O2—Cu	126.58 (12)	C11—C10—H10B	110.1
C3—O3—C4	116.84 (15)	H10A—C10—H10B	108.4
C6—O4—C7	117.37 (15)	N2—C11—C10	108.56 (15)
C14—O5—C15	116.82 (14)	N2—C11—H11A	110.0
C17—O6—C18	116.84 (15)	C10—C11—H11A	110.0
C9—N1—C10	120.01 (15)	N2—C11—H11B	110.0
C9—N1—Cu	126.26 (13)	C10—C11—H11B	110.0
C10—N1—Cu	113.69 (12)	H11A—C11—H11B	108.4
C12—N2—C11	120.56 (16)	N2—C12—C13	124.04 (16)
C12—N2—Cu	126.73 (13)	N2—C12—H12A	118.0
C11—N2—Cu	112.69 (12)	C13—C12—H12A	118.0
O1—C1—C2	117.14 (16)	C12—C13—C20	122.61 (16)
O1—C1—C8	123.79 (16)	C12—C13—C14	118.91 (16)
C2—C1—C8	119.07 (16)	C20—C13—C14	118.19 (16)
C3—C2—C1	120.24 (16)	O5—C14—C16	122.73 (16)
C3—C2—H2A	119.9	O5—C14—C13	115.18 (15)
C1—C2—H2A	119.9	C16—C14—C13	122.09 (17)
O3—C3—C2	123.78 (17)	O5—C15—H15A	109.5
O3—C3—C5	114.10 (16)	O5—C15—H15B	109.5
C2—C3—C5	122.12 (17)	H15A—C15—H15B	109.5
O3—C4—H4A	109.5	O5—C15—H15C	109.5
O3—C4—H4B	109.5	H15A—C15—H15C	109.5
H4A—C4—H4B	109.5	H15B—C15—H15C	109.5
O3—C4—H4C	109.5	C14—C16—C17	118.54 (17)
H4A—C4—H4C	109.5	C14—C16—H16A	120.7
H4B—C4—H4C	109.5	C17—C16—H16A	120.7
C6—C5—C3	118.34 (17)	O6—C17—C19	124.27 (17)
C6—C5—H5A	120.8	O6—C17—C16	113.88 (16)
C3—C5—H5A	120.8	C19—C17—C16	121.85 (16)
O4—C6—C5	123.59 (17)	O6—C18—H18A	109.5
O4—C6—C8	114.43 (16)	O6—C18—H18B	109.5
C5—C6—C8	121.97 (17)	H18A—C18—H18B	109.5
O4—C7—H7A	109.5	O6—C18—H18C	109.5
O4—C7—H7B	109.5	H18A—C18—H18C	109.5
H7A—C7—H7B	109.5	H18B—C18—H18C	109.5
O4—C7—H7C	109.5	C17—C19—C20	120.29 (17)
H7A—C7—H7C	109.5	C17—C19—H19A	119.9
H7B—C7—H7C	109.5	C20—C19—H19A	119.9
C9—C8—C6	118.82 (16)	O2—C20—C19	116.76 (16)
C9—C8—C1	122.92 (16)	O2—C20—C13	124.37 (16)
C6—C8—C1	118.23 (16)	C19—C20—C13	118.86 (16)
			
O2—Cu—O1—C1	160.64 (16)	Cu—N1—C9—C8	−3.0 (3)
N1—Cu—O1—C1	−14.77 (16)	C6—C8—C9—N1	175.14 (18)
O1—Cu—O2—C20	158.92 (16)	C1—C8—C9—N1	−6.6 (3)
O1—Cu—N1—C9	11.10 (17)	C9—N1—C10—C11	154.24 (17)
N2—Cu—N1—C9	−173.59 (17)	Cu—N1—C10—C11	−27.89 (19)
O1—Cu—N1—C10	−166.61 (13)	C12—N2—C11—C10	149.54 (18)
N2—Cu—N1—C10	8.70 (13)	Cu—N2—C11—C10	−32.21 (19)
O2—Cu—N2—C12	16.82 (17)	N1—C10—C11—N2	37.8 (2)
N1—Cu—N2—C12	−168.03 (17)	C11—N2—C12—C13	170.68 (17)
O2—Cu—N2—C11	−161.30 (13)	Cu—N2—C12—C13	−7.3 (3)
N1—Cu—N2—C11	13.86 (13)	N2—C12—C13—C20	−8.9 (3)
Cu—O1—C1—C2	−169.17 (13)	N2—C12—C13—C14	177.42 (18)
Cu—O1—C1—C8	10.4 (3)	C15—O5—C14—C16	−1.6 (3)
O1—C1—C2—C3	−179.68 (17)	C15—O5—C14—C13	178.48 (16)
C8—C1—C2—C3	0.8 (3)	C12—C13—C14—O5	−8.4 (2)
C4—O3—C3—C2	−0.5 (3)	C20—C13—C14—O5	177.63 (15)
C4—O3—C3—C5	180.00 (17)	C12—C13—C14—C16	171.67 (17)
C1—C2—C3—O3	178.78 (17)	C20—C13—C14—C16	−2.3 (3)
C1—C2—C3—C5	−1.8 (3)	O5—C14—C16—C17	178.48 (16)
O3—C3—C5—C6	−179.16 (17)	C13—C14—C16—C17	−1.6 (3)
C2—C3—C5—C6	1.3 (3)	C18—O6—C17—C19	7.5 (3)
C7—O4—C6—C5	13.4 (3)	C18—O6—C17—C16	−172.08 (17)
C7—O4—C6—C8	−166.65 (16)	C14—C16—C17—O6	−176.78 (16)
C3—C5—C6—O4	−179.92 (17)	C14—C16—C17—C19	3.7 (3)
C3—C5—C6—C8	0.1 (3)	O6—C17—C19—C20	178.80 (17)
O4—C6—C8—C9	−2.7 (3)	C16—C17—C19—C20	−1.7 (3)
C5—C6—C8—C9	177.33 (18)	Cu—O2—C20—C19	−174.25 (12)
O4—C6—C8—C1	179.00 (16)	Cu—O2—C20—C13	7.1 (3)
C5—C6—C8—C1	−1.0 (3)	C17—C19—C20—O2	178.98 (17)
O1—C1—C8—C9	2.8 (3)	C17—C19—C20—C13	−2.3 (3)
C2—C1—C8—C9	−177.69 (17)	C12—C13—C20—O2	9.1 (3)
O1—C1—C8—C6	−178.94 (17)	C14—C13—C20—O2	−177.20 (17)
C2—C1—C8—C6	0.6 (3)	C12—C13—C20—C19	−169.52 (17)
C10—N1—C9—C8	174.61 (17)	C14—C13—C20—C19	4.2 (3)

### Hydrogen-bond geometry (Å, °)

**Table:**

*D*—H···*A*	*D*—H	H···*A*	*D*···*A*	*D*—H···*A*
C18—H18A···O5^i^	0.98	2.57	3.439 (2)	148

Symmetry codes: (i) *x*, −*y*+1/2, *z*−1/2.
